# Image Inversion during Xi Robotic ventral hernia repair: making it even more effective

**DOI:** 10.1590/0100-6991e-20202879

**Published:** 2021-03-17

**Authors:** ANDRE LUIZ GIOIA MORRELL, ALEXANDER CHARLES, ALLAN GIOIA MORRELL, JOSE MAURICIO FREITAS MENDES, ALEXANDER MORRELL

**Affiliations:** 1 - Instituto Morrell, Cirurgia do Aparelho Digestivo Minimamente Invasiva e Robótica - São Paulo -SP - Brasil; 2 - Sociedade Beneficente Israelita Brasileira Albert Einstein, Cirurgia Geral e do Aparelho Digestivo Minimamente Invasiva e Robótica - São Paulo - SP - Brasil; 3 - Rede D’Or São Luiz, Cirurgia Geral e do Aparelho Digestivo Minimamente Invasiva e Robótica - São Paulo - SP - Brasil; 4 - Hospital Vila Nova Star, Cirurgia Geral e do Aparelho Digestivo Minimamente Invasiva e Robótica - São Paulo - SP - Brasil; 5 - Hospital Leforte, Cirurgia Geral e do Aparelho Digestivo Minimamente Invasiva e Robótica - São Paulo - SP - Brasil

**Keywords:** Robotics, Hernia, eTEP, Ventral Hernia, Image Inversion, Hérnia, Cirurgia Robótica, Hérnia Ventral, eTEP

## Abstract

**Introduction:**

currently, there are several clinical applications for robot-assisted surgery and in the hernia scenario, robot-assisted surgery seems to have the ability to overcome laparoscopic ventral hernias repairs limitations, facilitating dissection, defect closure, and mesh positioning. Exponentially grown in numbers of robotic approaches have been seen and even more complex and initially not suitable cases have recently become eligible for it. An appropriate tension-free reestablishment of the linea alba is still a major concern in hernia surgery and even with the robotic platform, dissecting and suturing in anterior abdominal wall may be challenging. This article reports a technical image artifice during a da vinci Xi-platform robotic ventral hernia repair allowing the surgeon to establish a more familiar and ergonomic manner to perform dissection and suturing in anterior abdominal wall.

**Technical Report:**

a step by step guided technique of image inversion artifice is described using detailed commands and figures to assure optimal surgical field and ergonomics whenever acting in robotic ventral hernias repair with the da Vinci Xi-platform. Our group brief experience is also reported, showing an easy and reproducible feature among surgeons with safe outcomes*.*

**Conclusion:**

we consider that image inversion artifice is a simple and reproducible feature in robotic ventral hernia repair. Through a step-by-step guide, this report enables the creation of an artifice providing a comfortable operative field and allowing the surgeon to achieve its best proficiency in hernia surgery.

## RESUMO

**Introdução:**

as qualidades da cirurgia robótica em diversos campos da cirurgia minimamente invasiva são notórias e, no cenário de hérnias abdominais, ela tem se mostrado capaz de superar as limitações da laparoscopia facilitando a dissecção, o fechamento do defeito herniário e o posicionamento da tela. O número de abordagens robóticas cresceu exponencialmente e casos ainda mais complexos e inicialmente inadequados tornaram-se recentemente elegíveis para uma cirurgia menos invasiva. A reconstrução adequada da linha alba e livre de tensão ainda é uma grande preocupação na cirurgia de hérnia e, mesmo com a plataforma robótica, dissecar e suturar na parede abdominal anterior pode ser um desafio. Este artigo relata um artifício técnico com inversão de imagem durante a correção de hérnia ventral robótica com a plataforma Da vinci Xi, permitindo ao cirurgião estabelecer uma maneira mais familiar e ergonômica de realizar dissecção e sutura na parede abdominal anterior.

**Nota Técnica:**

uma técnica guiada passo a passo com artifício de inversão de imagem é descrita usando comandos e figuras detalhadas para garantir campo cirúrgico ideal e melhor ergonomia ao cirurgião sempre que atuar na correção de hérnias ventrais robóticas com a plataforma Da vinci Xi. Nossa breve experiência de grupo também é relatada, mostrando-se uma técnica fácil e reprodutível entre cirurgiões com resultados seguros.

**Conclusão:**

consideramos que o artifício de inversão de imagens é uma característica simples e reprodutível na correção de hérnia ventral robótica. Por meio de um guia passo a passo, este artigo detalha a criação de um artifício técnico que proporciona um campo operatório confortável ao cirurgião atingindo sua melhor proficiência em cirurgia de hérnia.

## INTRODUCTION

Ventral hernia repair (VHR) is one of the most common operations performed by general surgeons^1^. The optimal technical approach to this condition is still in perspective having the majority of these elective repairs carried out through conventional midline approach^2^. Due to high incidence of wound morbidity associated with open hernia repairs, the laparoscopic approach was initially seen as a possible technique to decrease these complications^3^. 

Minimally invasive surgery has revolutionized surgical treatment of diseases for a variety of pathologic conditions with shortened hospitalizations, less pain, decreased recovery time, faster return to activity, improved cosmesis, and reduction in wound morbidity. Specific to VHR, laparoscopic approach revealed less surgical site infections and wound morbidity. However, recurrence rates were not negligible, ranging from 7 to 18%^4^ ^-^ ^6^. The increased recurrence rate seen in laparoscopic surgery is likely due to the degree of technical difficulty to close the midline fascia, creating an appropriate large mesh overlap despite the big learning curve. 

Da Vinci robotic surgical system has brought huge evolution to minimally invasive surgery, and also improved the development of hernia procedures. Robotic technology seems to overcome some limitations of laparoscopy in restoring abdominal wall integrity. In fact, robotic surgery offers the advantages of several degrees of freedom, 3D imaging, stable camera and superior ergonomics, which enable precise suturing and dissection at difficult angles^7^. In the robotic scenario for VHRs, a variety of techniques such as transabdominal preperitoneal repair (TAPP), intraperitoneal onlay mesh with or without fascial closure (IPOM plus and IPOM) and totally extraperitoneal (eTEP) added to posterior components separation techniques (TAR) can be ordinarily accomplished. Popularized by Belyansky et al.^8^, the enhanced-view totally extraperitoneal (eTEP) robotic ventral hernia repair technique turned out to be a game changer in abdominal wall surgery. Even larger defects initially thought not to be eligible for a minimally invasive surgery showed off encouraging safety and feasibility results. Stimulated by its outcomes, bigger defects and more complex abdominal wall hernias have been addressed to robotic approach and its benefits are in perspective^9^. 

**Figure 1:**
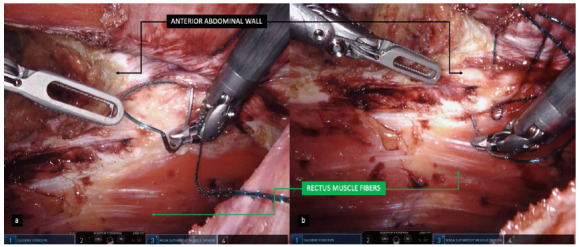
A: Image with the perspective of the anterior abdominal wall in the upper quadrant of the surgeon display, acting in the “ceiling” / B: Visualization of a less comfortable and usual layout when approaching defects of the anterior abdominal wall.

**Figure 2:**
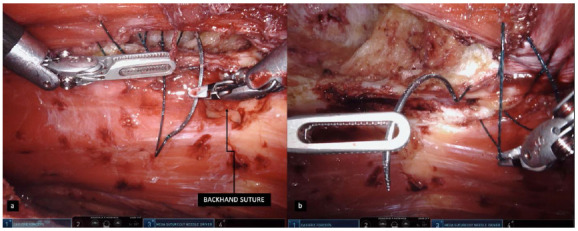
A and B: Suture performed with pronation movement in a backhand maneuver, less comfortable and slower when compared to the forehand supination movement.

Whenever performing a VHR, one of the most important steps of the procedure is achieving a midline closure. It is common sense that surgeons are more familiarized to an anterior to posterior perspective of analyzing structures, looking down in the abdominal cavity, and performing suturing since endoscopes and trocars are often positioned in the anterior abdominal wall. However, in VHR, due to the anterior position of the defect from the endoscope and trocars perspective, normally positioned lateral or in an inferior or superior topography of the abdomen, suturing is more challenging than usual practice making the surgeon looking up (Figure 1). Also, a forehand suturing commonly used to bring structures together is substituted for a backhand suturing, known to be more difficult and with higher mean time of performance^10^ (Figure 2). Therefore, the purpose of this article is to point out and describe a technical artifice during robotic ventral hernia repair in the Xi platform to achieve a digital image inversion feature (INV), enabling the surgeon to perform any suturing in an upside-down perspective added to a forehand old fashion disposal. By achieving this maneuver, a more comfortable and familiar suturing is achieved allowing more efficiency in less surgical time.

## METHODS

This article reports a surgical feature in a standardization step-by-step description for robotic expert surgeons whenever performing a robotic ventral hernia repair. Also, a retrospective review of a prospective maintained database of this surgical technique was done between June 2018 and August 2020, showing its results. Patients who underwent robotic ventral hernia repairs using Da Vinci Xi platforms were analyzed, excluding all laparoscopic or Da Vinci Si robotic ventral hernia procedures. Data was collected including demographics, preoperative, intraoperative and postoperative variables.

### Technical Report

The procedure involves using a 4-arm standard Da Vinci XI surgical system (Da Vinci Surgical System; Intuitive Surgical, Inc., Sunnyvale, CA). This artifice can be achieved independent of the robot docking setup as well as surgical technique approach, whether transperitoneal or extraperitoneal. Not different from a usual Xi robotic-assisted procedure, port placement should include a 30° endoscope robotic endoscope and robotic instruments. The image inversion artifice (INV) can be achieved at any time during the procedure depending on the surgeon’s necessity through a sequence of maneuvers on both the console and patient cart robotic instruments.

### Bedside steps

On bedside, four sequential steps should be done by the bedside assistant surgeon to assure image inversion correctly. First, the instruments must be switched to opposite arms. The needle driver normally disposed of in the right hand of a right-handed surgeon should be positioned in the left arm and the Cadiere or bipolar forceps on the right arm. Second, by tapping the robotic camera arm clutch button, the robotic camera is allowed to be free of rotational movement. Third step is achieved by inverting in a 180° rotation the robotic endoscope, making it disposed upside down, without undocking the camera from the robotic arm. Fourth and last bedside step is done by clutching again the button in the robotic camera arm, making the endoscope ready for surgeons use.

### Surgeons console steps

**Figure 3:**
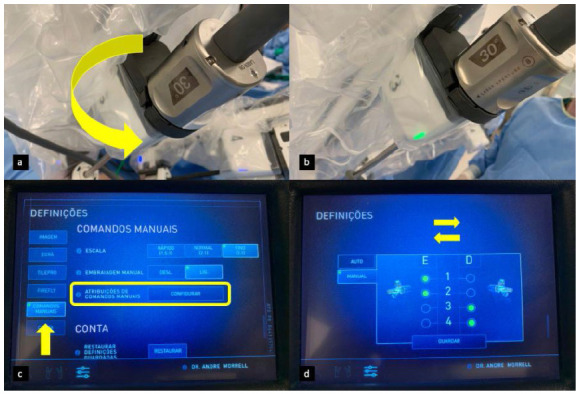
A: Endoscope disposed with 30 degrees looking up, before rotation / B: Endoscope after rotation, disposed with 30 degrees looking down / C and D: Display at the console to access instruments reassignment.

Using the display at the robotic console, more three steps must be done to enable INV feature. First step is performed by reassigning robotic instruments to opposite hands. Display should be unlocked, followed by a click in the “manual command” button, which will display the button to get the reassignment of the robotic arms shown in Figure 3 (Figure 3c, d). After doing it, the second step is done by taping the swap pedal to confirm the new configuration. The third and final step is then fulfilled by flipping the endoscope 30° lens in the opposite direction. 

**Figure 4:**
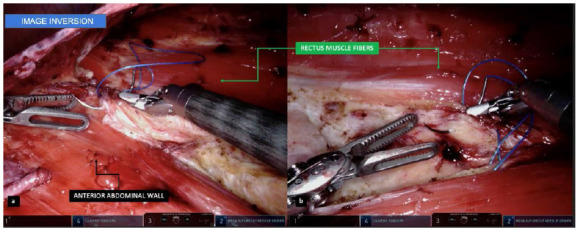
A: Visualization generated with the image inversion artifice, allowing a surgical field in the lower corner of the screen, acting on the “floor”. B: Handling the structures and handling the needle in its most comfortable form.

**Figure 5:**
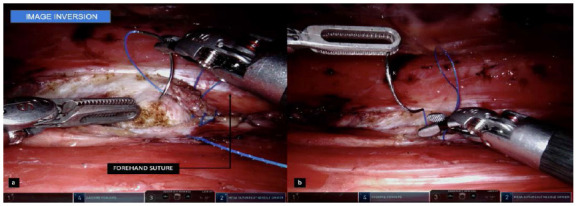
A: Performing a suture using supine forehand movements, more ergonomic, comfortable and habitual for the surgeon when compared to pronation of the backhand wrist.

**Figure 6:**
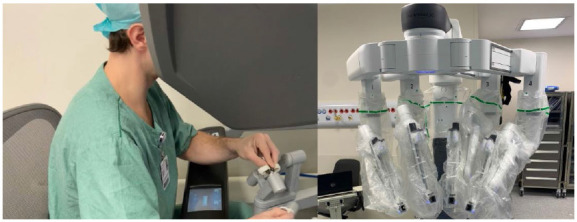
da Vinci Xi platform and surgeons console.

By completing these both bedside and surgeon’s console guided steps, an image inversion artifice is generated resulting in any movement that would previously be performed in the upper space of the surgical field (“ceiling”) is transformed into movements in the lower field (“floor”), more familiar and effective and easily done in a forehand suture (Figure 4, 5, 6).

## RESULTS

A total of nineteen patients were operated on using this image inversion artifice method between June 2018 and August 2020. Surgery indications were all due to abdominal ventral hernias, with five of them presenting primary ventral hernias (26.3%) and fourteen patients with incisional defects (73.7%). Total mean age was 47.4 years (range: 35-57 years) with a mean BMI of 28.4 kg/m2. Mean defect greatest dimension size was 6.2 cm (range: 3.5-11 cm). Robotic docking was lateral in all cases and performed with the Vinci Xi platform. All surgical procedures were performed with the eTEP access technique. Mean console time was 142.2 (range: 90-210 min) and image inversion artifice steps were mainly performed in 40 seconds. Restoration of anterior abdominal wall and linea alba were achieved in all cases by a comfortable and forehand suture. No intraoperative complications occurred neither conversion to lap or open procedures. Postoperative was uneventful having no wound morbidity or recurrence rate within the median follow-up period of 410.1 days (range: 88 - 724 days). 

## DISCUSSION

Although initial laparoscopic VHR decreased wound morbidity, it didn’t grant an optimal fascial defect closure with adequate tissue approximation^11^. Restoring abdominal wall integrity by closure of the fascia is an extremely important factor during VHR with couple studies reporting higher rates of recurrence when failing its accomplishment^12^. Superior outcomes of robotic surgery have been clearly demonstrated for some procedures, most notably in gynecologic and urologic fields, but its applicability to general surgery grows substantially. Although recent, robotic-assisted surgeries have addressed interesting applications in the abdominal wall^13^. Robotic inguinal hernia repairs showed encouraging short-term outcomes even in former surgeons’ hands^14^. In the field of ventral herniorrhaphy, high definition endoscope, naked eye 3D imaging system and endowrist instruments makes Da Vinci robotic system feasible to dissect and complete the suture in the anterior abdominal despite its unusual perspective and needle handling. 

Due to encouraging outcomes, the number of robotic ventral hernia repairs has grown exponentially in the last 2 years worldwide. Headed by Beliansky et al.^8^, surgeons started performing robotic VHR in several approaches and even in more complex cases, initially thought not to be suitable. A comparative review study described favorable perioperative outcomes and low recurrence rates undergoing robotic VHR, especially in morbid obese patients and more complex abdominal wall defects^15^. With regards to robotic eTEP access, Kudsi and Gokcal^16^ also described fifty-two patients submitted to an exclusively lateral approach with and without transversus abdominis release (TAR) technique showing positive postoperative results. In Brazil, the first and biggest case-series in Latin-America has recently been reported by Morrell et al.^17^, with seventy-four patients undergoing robotic eTEP VHR without conversion rates or hernia recurrence. 

The gold standard parameter of any hernia repair is recurrence rate. Open and laparoscopic repairs carry not negligible recurrence rates of 32% and up to 18% in the literature, respectively^18^ ^,^ ^19^. Many studies have shown RVHR to be durable in the short term with recurrence rates < 1%^8^ ^,^ ^16^ ^,^ ^17^ ^,^ ^20^. One of the clear advantages of robotic VHR is the possibility for posterior component separation to be performed in more complex cases with lower morbidity and hospital length of stay^21^. 

Cost is a frequently cited deterrent to the application of robotics to general surgery, which is often related to an initial higher capital expense with the robotic system. In a more efficient long-term scenario, any feature enabling surgeons to be faster and safer could bring better results and save costs with lesser operative rooms time and hospitalization length, better outcomes as well as lower readmissions rates. The image inversion artifice (INV) is a simple, fast, and reproducible tool in VHR. This step-by-step guided report provides the surgeon a clear orientation on how to exactly perform the digital feature intraoperatively that could bring a more comfortable scenario allowing the surgeon to achieve its best proficiency. Therefore, an image inversion artifice in the robotic ventral hernia repair combines the benefits of decreased morbidity of the minimally invasive techniques with a comfortable and durable tension-free midline fascial closure seen during open surgery.

## CONCLUSION

This present study remains consistent with the published literature determining the current aspects of robotic ventral hernia repair. In addition, this article presents and describes a step-by-step guide to an unprecedent technical feature during robotic ventral hernia repair in the Xi platform to achieve a digital image inversion artifice (INV). We consider that a simple and fast artifice may contribute to a more familiarized and efficient restoration of abdominal wall integrity whenever approximating anterior midline defects.
